# Acinar phenotype is preserved in human exocrine pancreas cells cultured at low temperature: implications for lineage-tracing of β-cell neogenesis

**DOI:** 10.1042/BSR20150259

**Published:** 2016-05-06

**Authors:** Josué K. Mfopou, Isabelle Houbracken, Elke Wauters, Iris Mathijs, Imane Song, Eddy Himpe, Jonathan Baldan, Harry Heimberg, Luc Bouwens

**Affiliations:** * Cell Differentiation Unit, Diabetes Research Center, Vrije Universiteit Brussel (VUB), Brussels, BE 1090, Belgium; † Beta Cell Neogenesis, Diabetes Research Center, Vrije Universiteit Brussel (VUB), Brussels, BE 1090, Belgium

**Keywords:** acinar cell, β-cell, chilled, hypothermic, lineage tracing, neogenesis, transdifferentiation

## Abstract

The regenerative medicine field is expanding with great successes in laboratory and preclinical settings. Pancreatic acinar cells in diabetic mice were recently converted into β-cells by treatment with ciliary neurotrophic factor (CNTF) and epidermal growth factor (EGF). This suggests that human acinar cells might become a cornerstone for diabetes cell therapy in the future, if they can also be converted into glucose-responsive insulin-producing cells. Presently, studying pancreatic acinar cell biology *in vitro* is limited by their high plasticity, as they rapidly lose their phenotype and spontaneously transdifferentiate to a duct-like phenotype in culture. We questioned whether human pancreatic acinar cell phenotype could be preserved *in vitro* by physico-chemical manipulations and whether this could be valuable in the study of β-cell neogenesis. We found that culture at low temperature (4°C) resulted in the maintenance of morphological and molecular acinar cell characteristics. Specifically, chilled acinar cells did not form the spherical clusters observed in controls (culture at 37°C), and they maintained high levels of acinar-specific transcripts and proteins. Five-day chilled acinar cells still transdifferentiated into duct-like cells upon transfer to 37°C. Moreover, adenoviral-mediated gene transfer evidenced an active Amylase promoter in the 7-day chilled acinar cells, and transduction performed in chilled conditions improved acinar cell labelling. Together, our findings indicate the maintenance of human pancreatic acinar cell phenotype at low temperature and the possibility to efficiently label acinar cells, which opens new perspectives for the study of human acinar-to-β-cell transdifferentiation.

## Abstract (precis)

*In vitro* cultured pancreatic acinar cells rapidly differentiate. Low temperature exposure prevents this process and improves the efficiency of acinar cell labelling with adenovirus vectors. This may help in tracing β-cell neogenesis from human pancreatic acinar cells.

## INTRODUCTION

Type 1 diabetes mellitus is a leading metabolic disease that might benefit from the ongoing cell therapy developments. Because its underlying pathophysiology involves a critically reduced β-cell mass within the pancreas, transplantation of the β-cells isolated from cadaveric donor pancreata is the current unique method to restore insulin-independence in patients. However, only a limited number of selected patients effectively undergo β-cell therapy owing to the severe shortage in donor pancreata [1]. This situation prompted investigators to explore alternative sources of insulin-producing cells, which encompass somatic cells from the pancreas or other tissues, and multipotent or pluripotent stem cells [1,2]. Within the pancreas, a particular attention is paid to acinar cells because of their high abundance and their differentiation plasticity [3,4].

Isolated exocrine cells from rodent and human pancreas rapidly down-regulate acinar markers (Amylase, Elastase, Chymotrypsin, Ptf1a (pancreas transcription factor 1 subunit alpha) and Mist1) and undergo dedifferentiation to progenitor-like cells or transdifferentiation to ductal-like cells with features of epithelial-mesenchymal transition [4–8]. Interestingly, it was shown that transdifferentiated acinar cells from rodent pancreas can be induced to generate new β-cells *in vitro*, and that this requires similar molecular signals as during embryonic pancreas development [9–12]. In addition, transduction of different combinations of neurogenin 3 (Ngn3), MafA and pancreatic and duodenal homeobox 1 (Pdx1) in acinar cells leads to the controlled derivation of δ-, α- or β-cells [13]. Furthermore, when mouse acinar cells overexpressing the thyroid hormone receptor α are treated with thyroid hormone, they activate the above-mentioned transcription factors and differentiate into β-cells [14].

*In vivo*, mouse acinar cells could also be induced to differentiate into β-cells by combined overexpression of the transcription factors Ngn3, MafA and Pdx1 [15]. More importantly, transgenic mice rendered chronically diabetic by the β-cell toxin alloxan and treated with both ciliary neurotrophic factor (CNTF) and epidermal growth factor (EGF) regenerate new β-cells from labelled acinar cells within the pancreas [16]. This latter study suggests that the generation of autologous β-cells could be endogenously induced from the human pancreatic exocrine compartment to cure diabetes.

It is however compulsory that these observations in rodents are reproduced with human pancreatic acinar cells, in order to really consider them as a potential β-cell source for treating diabetes patients. One difficulty in revealing human acinar-to-β-cell conversion *in vitro* lies in the phenotypic instability of these cells. Indeed, rapid down-regulation of acinar cell-specific genes precludes the use of genetic labelling; whereas non-genetic methods are usually not optimal for long-term tracing. Previous studies recommended lentiviral vectors for labelling rat pancreatic acinar cells *in vitro* [17], but the need for genome integration before reporter expression precludes its use for optimally tracing acinar cells since specific marker genes are rapidly silenced in culture. We assume that this limitation could be overcome by methods that can stabilize acinar cell phenotype *in vitro*. To this end, few reports showed that Matrigel-embedded pancreatic slices or pancreas outgrowths cultured at the gas–liquid interphase maintain the acinar cell phenotype for about 4 days [18–20]. Although interesting, these models probably do not allow for optimal lineage tracing because of the material thickness that limits gene transfer efficiency.

Only one previous report by Coutts et al. [21] showed that hypothermic storage could help isolated human pancreatic acinar cells to keep their original phenotype for 7 days in a basal culture medium; but analysis was restricted to detection of few acinar-specific proteins. We questioned whether hypothermic stored pancreatic exocrine fraction continued to express acinar cell-related transcripts, and whether these properties could be used for tracing. The effect of low temperature was therefore studied on human pancreas exocrine fraction, which is obtained after islet isolation for diabetes cell therapy. Although the temperature was considered as the major determinant of our observations, we cannot rule out the influence of different CO_2_/O_2_ levels on chilled cells. Nevertheless, our findings reproduced those of Coutts et al. [21], and indicated that low temperature culture preserves acinar cell morphological and molecular features for up to 10 days. In these settings, acinar cells did not silence the promotors of several specific marker genes, and they moderately activated transcription of the pro-endocrine gene *NGN3*. We used adenoviral reporters to demonstrate improved labelling of chilled human acinar cells, therefore delineating a culture system that may uncover human acinar-to-β-cell transdifferentiation in the future.

## MATERIALS AND METHODS

### Acinar cell culture and transduction

The human pancreatic exocrine cell fraction was obtained after islet isolation from the Beta Cell Bank of the Diabetes Research Center in Brussels. Cells were washed twice with Dulbecco's phosphate buffered saline (D-PBS) and allowed to sediment for 5 min. The cell mass brought to each culture condition was standardized by resuspending 100 μl of 5 min sedimented pellet into 10 ml culture medium. This cell suspension was transferred into non-adherent culture dishes and maintained at 37°C (control) or in the refrigerator (4–8°C, chilled) for 5–10 days. Whereas the CO_2_/O_2_ levels were controlled in the incubator, this was not the case for cultures at low temperature. We therefore cannot show evidence of the potential effects that the actual CO_2_/O_2_ levels in the refrigerator would have on our model, but we will assume that our findings is related to the temperature difference between both systems. The culture medium, which was refreshed every other day, consisted of Advanced RPMI (Life Technologies) supplemented with 1× penicillin–streptomycin solution (Sigma–Aldrich) and 5% FBS (HyClone). Before media change, cell clusters were allowed to sediment for 5 min at room temperature and the supernatant was discarded by suction.

In some experiments, 5-day-old control and chilled clusters were transferred from suspension dishes to tissue culture plates and further cultured at 37°C (secondary culture). To examine the activity of Amylase promoter in cultured cells, transduction was performed with two adenoviral vectors at a multiplicity of infection (MOI) of 100 each. These consisted of Ad-pAmy-CRE [4] and Ad-CMV-Lox-Stop-Lox-GFP (green fluorescent protein) [4], allowing for expression of the CRE recombinase in cells with an active Amylase promoter, followed by excision of the Lox-Stop-Lox site and thereby activating GFP expression. Control transductions were performed with Ad-CMV-GFP at MOI 100. An additional transduction assay was also performed with the lentiviral vector Le-CMV-eGFP at MOI 100. Except when otherwise stated, transduction was performed for 3 h in Advanced RPMI supplemented with 1× penicillin–streptomycin solution, then the viral vectors were washed away and the cells further cultured as described above.

### Immunofluorescence and microscopy

An initial sample was harvested on the day of isolation, fixed in 4% formaldehyde (Labonord) for 1 h, and thoroughly rinsed with PBS. Additional samples from control or chilled cells were harvested on day 1, 3 and 5 during culture and also fixed in 4% formaldehyde. All samples were further processed and sectioned as previously described [23]. Upon dehydration and blockade of non-specific binding sites in 2% naïve donkey serum (Jackson ImmunoResearch Laboratories) for 30 min, sections were incubated overnight at 4°C with dilutions of the primary antibodies in combination: rabbit anti-Amylase (1/400; A8273, Sigma–Aldrich), rabbit anti-Chymotrypsin (1/500; Santa Cruz Biotechnology), mouse anti-CK19 (cytokeratin 19) (1/50; M0888; Dako), guinea-pig anti-Insulin (1/3000; Prof C.V. Schravendijck, VUB, Belgium) and goat anti-PDX1 (1/200; AF2419, R&D Systems). Sections were washed thrice in PBS and incubated with the corresponding FITC- or TRITC-labelled secondary antibodies (all from Jackson ImmunoResearch Laboratories) for 1 h at room temperature. DNA in the cell nuclei were stained with Hoescht added together with the secondary antibody dilutions. Sections were finally rinsed thrice in PBS and mounted with a glass slide using Vectashield mounting medium (Vector Laboratories). In the experiments where cells were allowed to adhere and spread, samples were fixed directly in tissue culture plates for 15 min and kept in PBS until analysis. Except for a permeabilization step for 20 min in 70% ice-cold methanol, the immunofluorescence procedure was similar to that performed with sectioned samples on slides.

Samples were analysed under the Nikon TE 2000 Eclipse inverted fluorescent microscope (Nikon) and pictures were captured with a cooled camera device using the Nikon Imaging System (NIS) Elements AR v3.22 software (Nikon). Filter settings were reproduced between experiments to minimize variability in exposure times. The ratio of marker positive area to DAPI-stained area was estimated with the aid of the Object Count tool of the NIS Elements software as previously described [24].

### Western blot

Freshly isolated or cultured exocrine cells (approximately 10^6^ cells) were harvested and washed with PBS, and subsequently lysed with 250 μl of radio immunoprecipitation assay (RIPA) buffer supplemented with 1/10 β-mercaptoethanol. The protein concentration was determined by the Bradford method and 50 μg was used for SDS gel electrophoresis. Upon blotting on to nitrocellulose membranes, samples were incubated overnight at 4°C with primary antibodies dilutions (rabbit anti-β-actin, 1/2000, 4967S, Cell Signalling Technology, Bioke; rabbit anti-Amylase, 1/1000, A8273, Sigma–Aldrich; mouse anti-CK19, 1/1000, M0888, Dako). Membranes were washed and exposed to the secondary antibodies (goat anti-rabbit, 1/1000, Sigma–Aldrich or goat anti-mouse, 1/1000, Santa Cruz Biotechnologies) at room temperature for 1h, and the detection was performed using the chemiluminescence method.

### Gene expression analysis

Samples were harvested on the day of isolation and on culture days 1, 3, 5 and 10. After a brief wash in PBS, total RNA was extracted using the GenElute mammalian total RNA extraction kit (Sigma–Aldrich). The concentration and quality of RNA were assessed using the NanoDrop-1000 spectrophotometer (NanoDrop). A reverse transcription reaction was set up from 500 ng to 750 ng of total RNA using the GoScript RT system (Promega). All cDNA were further subjected to amplification on a fast thermal cycler (ABI 7900HT; Life Technologies) using the GoTaq qPCR assay (Promega) for *Amylase*, *Elastase*, *Chymotrypsin*, *CK19*, *SOX9*, *Insulin*, *Glucagon*, *PTF1a* and *MIST1*; the Express SybrGreener kit (Life Technologies) for *PDX1*; or the TaqMan assay (Life Technologies) for *NGN3.* The primer sequences used for reverse transcription polymerase chain reaction (RT-PCR) are available in the supplementary material. The amplification data were analysed following the d*C*_t_ method and reported as a percentage of the housekeeping gene (cyclophilin A) level in the corresponding samples.

### Statistical analysis

All experiments were performed at least three times with independent pancreas donors, and a few RNA samples were collected in duplicate. The results for quantitative data are displayed as mean with S.D. Statistical analysis consisted of the Mann–Whitney test for two samples and Kruskal–Wallis test with Dunn's multiple comparisons for several samples (one-way ANOVA). These tests were performed using Prism 5 software (GraphPad Software). The significance levels of several tests are shown with standard codes (*P<0.05, **P<0.01, ***P<0.001).

## RESULTS

### Human exocrine pancreas cells maintain their morphology and protein content in low temperature (chilled) cultures

**Figure 1:**
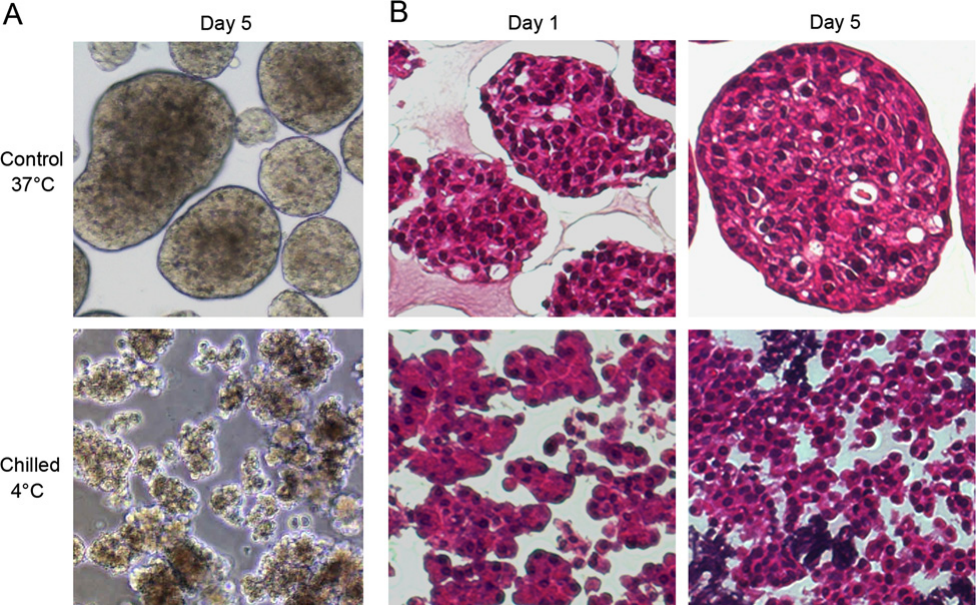
Human pancreatic acinar cell morphology in control and chilled cultures (**A**) Bright field images of acinar cells cultured at 37°C (control) or at 4–8°C (chilled) and pictured on day 5 after seeding. (**B**) Representative haematoxylin–eosin staining of the control and chilled acinar cells cultured for 1 day or 5 days. Note the absence of aggregate formation in chilled cultures.

Acinar cell preparations were cultured in the incubator (37°C, control) or in the refrigerator (4–8°C, chilled) for a few days and examined daily under the light microscope. As previously observed by Coutts et al. [21], human acinar cells in chilled cultures maintained the typical morphology coined to freshly isolated cells, as opposed to cells in control cultures that readily formed spherical aggregates of various sizes (Figure 1A). The divergence in cell morphology between the two conditions was already evident after culture for one day, and further increased with time spent in culture (Figure 1B). Whereas chilled cell clusters morphologically resembled fresh acinar clusters during the first week, those maintained for more than 10 days presented features of necrosis (results not shown) and were not further investigated.

**Figure 2:**
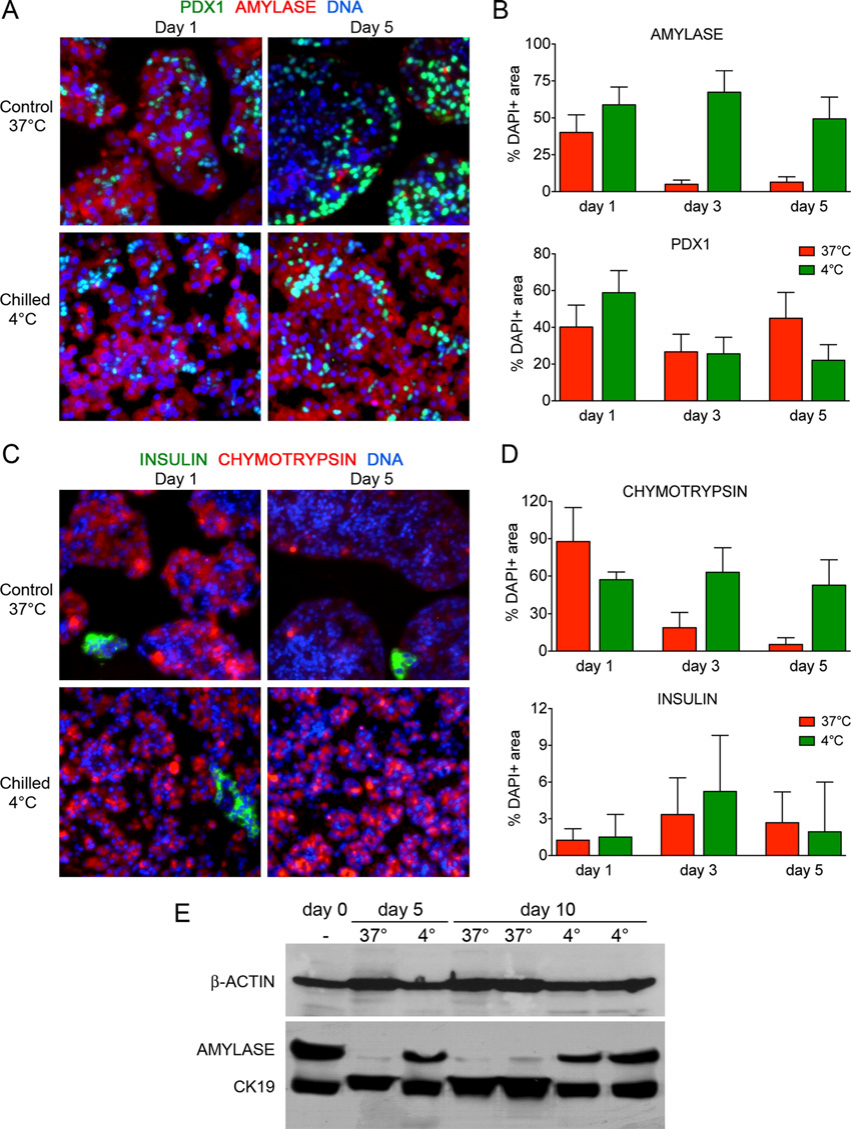
Protein detection in human pancreatic acinar cells cultured at 37°C or 4°C (**A**) Immunofluorescence analysis for Amylase and PDX1 in control and chilled cultures on day 1 and day 5. (**B**) Quantification of Amylase and PDX1 signals as a ratio to nuclei area identified by Hoescht staining. (**C**) Immunofluorescence analysis for Chymotrypsin and Insulin in control and chilled cultures on day 1 and day 5. (**D**) Quantification of Chymotrypsin and Insulin signals as a ratio to nuclei area identified by Hoescht staining. (**E**) Western blot detection of Amylase and CK19 in protein extracts collected on the day of isolation (day 0) or after 5 and 10 days culture. Note the striking reduction in Amylase signal at 37°C and the increased CK19 signal on the multiplex blot. β-Actin was used to control for protein loading.

We assessed the immunofluorescence detection of the acinar enzymes Amylase and Chymotrypsin in these cultures. Both proteins were present in the majority of freshly isolated exocrine cells (Supplementary Figures S1A and S1B), and also after chilled culture for 5 days. In contrast with chilled cells, clusters generated in control conditions showed very limited signals for these proteins (Figures 2A and 2C; Supplementary Figures S2A and S2B). A rough estimation of Amylase and Chymotrypsin abundances in both conditions is given as a ratio of positive area compared with the nuclei area. They indicate a progressive loss of Amylase (from 40.14 to 4.97% DAPI+ area) and Chymotrypsin (from 87.65 to 5.41% DAPI+ area) in control, which contrast with their maintenance in chilled cultures over 5 days (49.26–67.39% DAPI+ area for Amylase; 52.98–63.00% DAPI+ area for Chymotrypsin) (Figures 2B and 2D). The progenitor and β-cell marker PDX1 was detected in all samples and represented 22.07–58.84% of the nuclear area depending on the culture condition and time point (Figures 2A and 2B; Supplementary Figures S1B, S2A and S2B). The β-cell specific hormone Insulin was also detected (1.5–5% DAPI+ area), indicating the contamination by small islet fragments (Figures 2C and 2D). Most of the time, these islet fragments appeared necrotic in chilled cultures (Supplementary Figures S2A and S2B). We further confirmed the differential detection of Amylase and CK19 at 37°C and at 4°C by Western blot analysis of the corresponding protein extracts. Whereas Amylase was practically cleared off from extracts of cells maintained at 37°C, it remained abundantly detectable in chilled cell extracts up to day 10 (Figure 2E). Worthy to note, the ductal marker CK19 showed an opposite pattern. Together, these findings reproduce those of Coutts et al. [21] and indicate that human pancreatic acinar cells can be easily stored for several days in chilled conditions without a noticeable impairment in their morphological characteristics and presence of cell-specific proteins.

### Transcripts of exocrine, but not of endocrine genes remain detectable in chilled cultures

**Figure 3:**
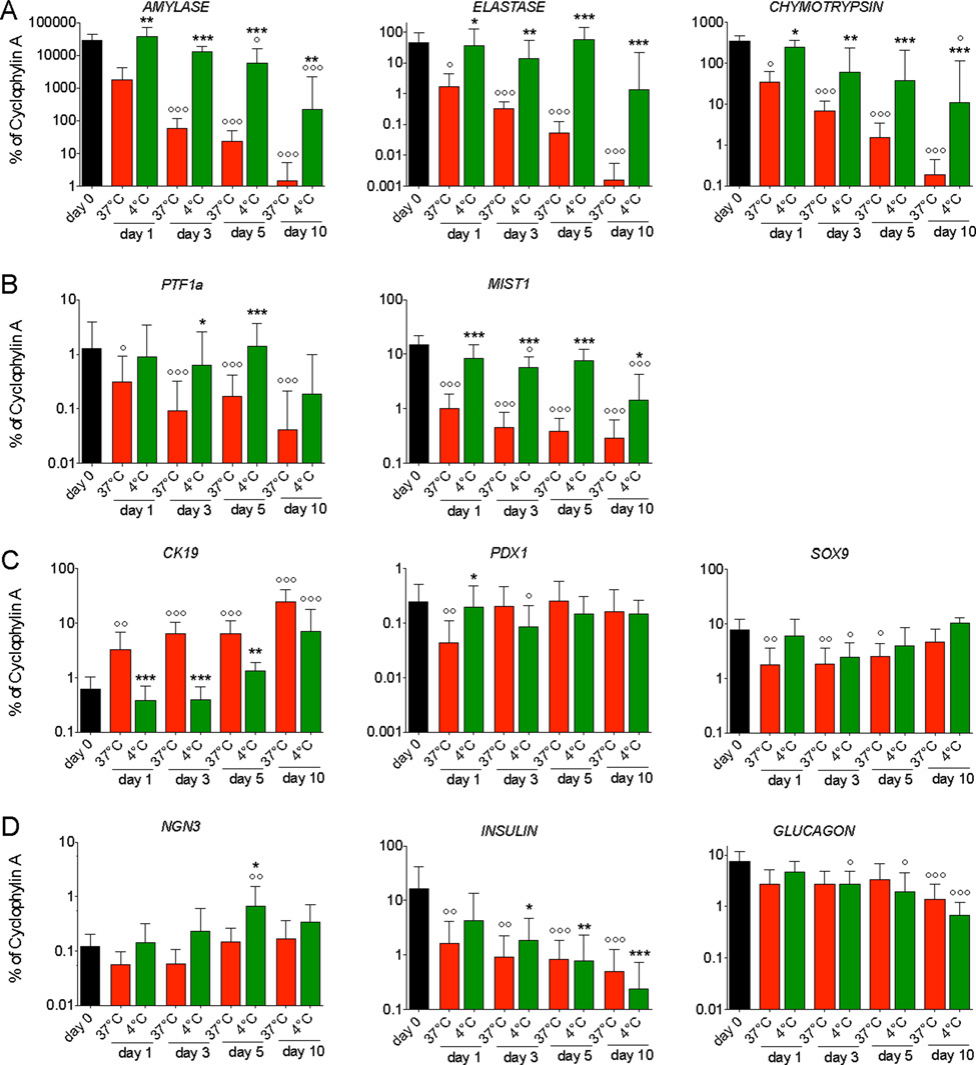
Transcripts profiling in control (37°C) and chilled (4°C) cultures of exocrine cells in comparison with the levels at isolation (day 0) (**A**) Transcripts of the acinar cell-specific enzymes are detected at high levels in chilled cells, whereas they are decreasing progressively in control cells. (**B**) The two acinar cell transcription factors *PTF1a* and *MIST1* follow a similar pattern as the enzymes. (**C**) Expression of *CK19* increases in both control and chilled cells, whereas *PDX1* and *SOX9* do not show a consistent profile. (**D**) A significant induction of *NGN3* expression is detected in chilled cells on day 5, in a context of decreasing *Insulin* and *Glucagon* transcripts. (° compared with day 0; * compared with 37°C culture)

We questioned whether the acinar cell-specific transcripts were still detectable after a few days of chilled culture. To this end, total RNA was collected upon isolation (day 0), after 1, 3, 5 or 10 days of control or chilled culture, and analysed by RT-PCR. We constantly detected transcripts of the three acinar cell-specific enzymes (*Amylase*, *Elastase* and *Chymotrypsin*) in chilled cultures for up to 10 days, and at levels that were usually comparable with freshly isolated exocrine cells (Figure 3A). In contrast, these markers were significantly and time dependently down-regulated in control cultures between isolation and culture day 10 (20000- to 30000-fold decrease for *Amylase* and *Elastase*, 2000-fold decrease for *Chymotrypsin*; Figure 3A). A similar pattern of gene expression was noticed for the acinar cell-specific transcription factors *PTF1a* and *MIST1*, which were rapidly and significantly lost in control (30- and 50-fold reduction respectively) but maintained in chilled cultures (Figure 3B).

In both control and chilled conditions, there was a steady increase in the transcript levels of the duct cell marker *CK19*, reaching respectively 40- and 10-fold on day 10. Remarkably, the expression of *CK19* was always significantly lower in chilled compared with control cells at all time points, and it was only after 10 days of chilled culture that *CK19* was significantly higher compared with the day of isolation. This suggests a delayed or restrained transdifferentiation process in chilled cultures (Figure 3C). On the contrary, the transcription factors *PDX1* and *SOX9*, which are expressed in pancreas progenitors and β-cells, and in ductal cells respectively, did not show any particular trend (Figure 3C; Supplementary Figures S1C, S2A and S2B).

To complete our gene expression profiling, we also assessed the expression of a few endocrine-related transcripts. We noticed a continuous reduction in *Insulin* and *Glucagon* transcripts in both control and chilled conditions between isolation and culture day 10. Unexpectedly, chilled cultures were consistently associated with a higher level of the pro-endocrine gene *NGN3*, with a maximal record on day 5 (6-fold compared with isolation day; Figure 3D). Therefore, chilled culture of human pancreatic exocrine cells is associated with a transcripts profile that significantly deviates from previous observations during acinar cell transdifferentiation *in vitro* (Supplementary Table S1).

### Chilled acinar cells undergo transdifferentiation in secondary cultures

**Figure 4:**
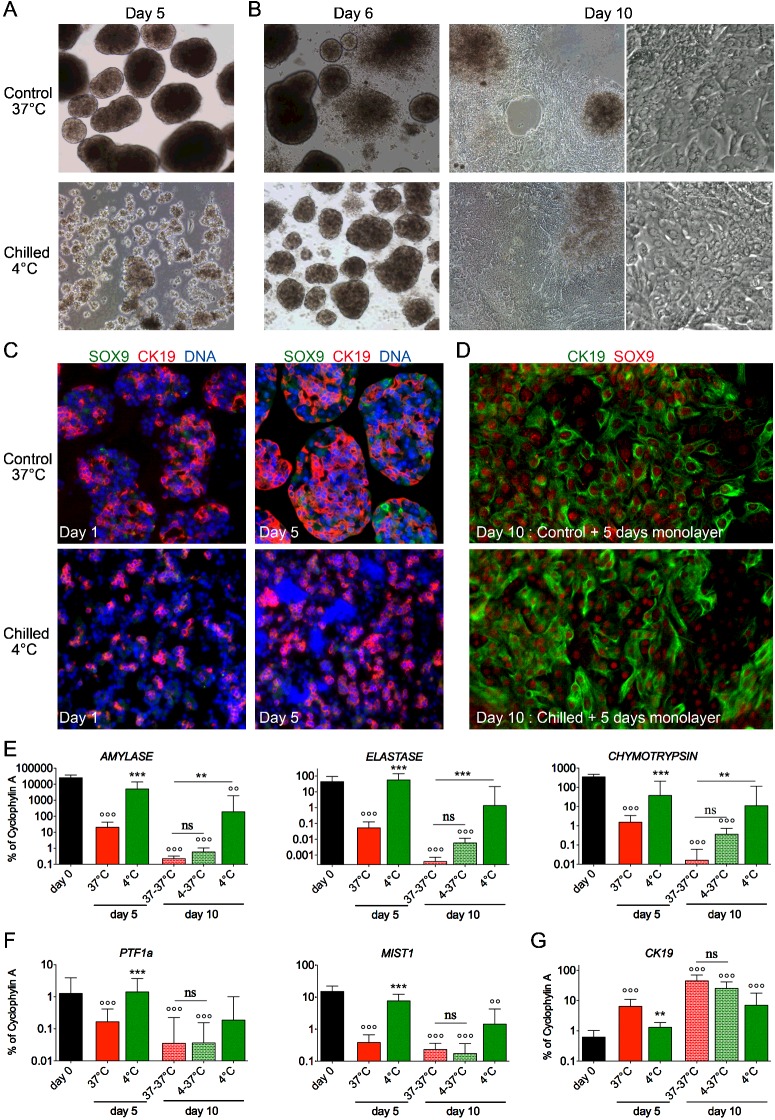
Transdifferentiation of exocrine cells in secondary cultures (**A** and **B**) Cell morphology after 5 days of suspension culture at 37°C or 4°C (A); and after adherence and spreading in tissue culture plates for 1 or 5 more days (day 6 and day 10 respectively) ; (B). Note that the monolayers formed in control are not distinguishable from those in chilled conditions. (**C** and **D**) Time-dependent expression of the duct cell markers CK19 and SOX9 during primary suspension (C) and secondary monolayer (D) culture. Expression of these markers increase in both control and chilled conditions. (**E**, **F** and **G**) Gene expression analysis in secondary cultures, showing down-regulation of acinar cell-specific enzymes (E) and transcription factors (F), and up-regulation of the duct cell gene *CK19* (G) during transdifferentiation in secondary cultures.

We next examined the potential of 5-day chilled acinar cells to engage in a transdifferentiation programme when returned in control culture conditions [4,7]. As previously described, during the first 2–3 days of suspension culture at 37°C, acinar cells consistently formed spherical clusters of various sizes (Figures 1A and 4A). Upon seeding in tissue culture plates on day 5 (secondary culture), these aggregates readily attached and spreaded out, forming a rough monolayer culture interspaced with small clumps. Interestingly, when 5-day chilled acinar cells were shifted to 37°C in tissue culture plates, clusters were rapidly formed from day 6, followed by attachment and spreading. They also produced a monolayer similar to the one obtained with control cells (Figure 4B).

The monolayers generated with these two approches essentially consisted of CK19+ and SOX9+ cells, suggesting that acinar cells from control and chilled primary cultures underwent acino-ductal transdifferentiation as previously described (Figures 4C and 4D) [4,7]. In contrast with freshly isolated exocrine cells or of cells chilled for a few days, only scarce and faint Amylase+ cells were identified in the secondary cultures (Supplementary Figures S1A, S1D, S1E and S2B). These findings were concordant with RT-PCR data that showed loss of acinar cell-specific genes (*Amylase, Elastase, Chymotrypsin*, *PTF1a* and *MIST1*) and up-regulation of the ductal marker *CK19* (Figures 4E and 4G) upon transdifferentiation of chilled exocrine cells in secondary culture. Worthy to note, the levels of acinar cell-specific transcripts were slighty higher (not significantly) after secondary culture of chilled cells as compared with the levels in control cells, which is concordant with their differentiation for only 5 days compared with 10 days (Figure 4E). The opposite also holds for ductal marker *CK19* (Figure 4G).

### The Amylase promoter in acinar cells can still be activated after 7-day chilled culture

**Figure 5:**
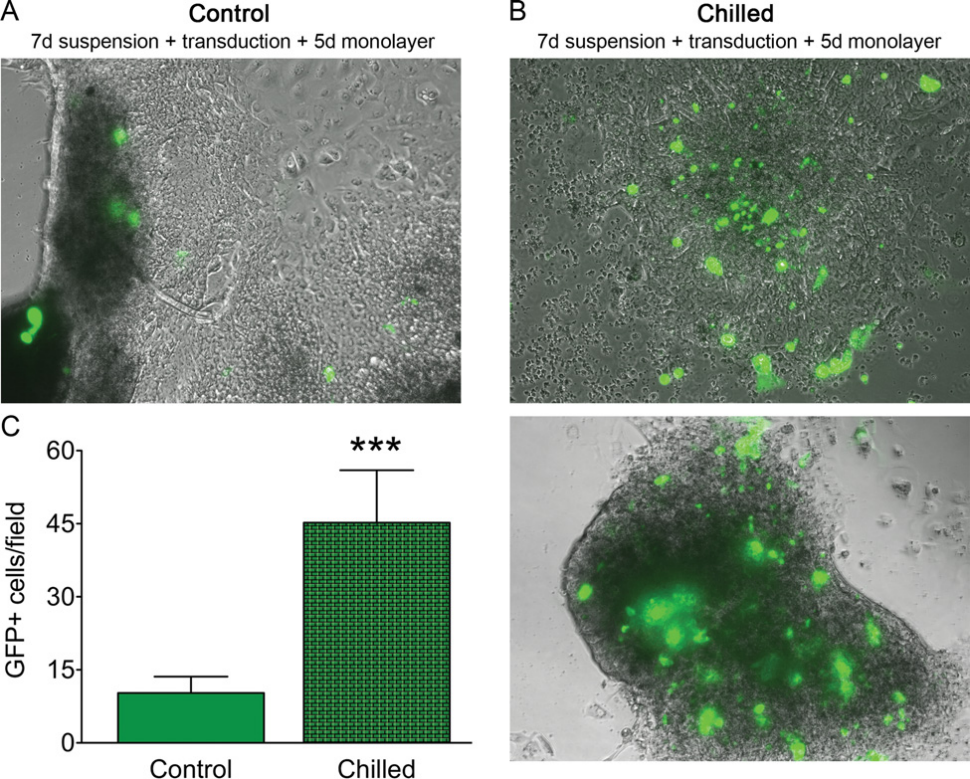
The Amylase promoter remains in active state in chilled acinar cells (**A**) Expression of GFP in control day 7 cells transduced with Ad-pAmy-CRE and Ad-CMV-Lox-Stop-Lox-GFP on day 7 and further cultured for 5 days as monolayers. (**B**) Representative fields showing GFP expression in day 7 chilled cells transduced with Ad-pAmy-CRE and Ad-CMV-Lox-Stop-Lox-GFP on day 7 and further cultured for 5 days as monolayers. (**C**) Quantification of GFP+ cells per field in control and chilled human pancreatic acinar cells transduced as described above.

Previous studies in our group indicated that acinar cells are not efficiently labelled with reporter transgenes because of the rapid loss of specific transcription *in vitro* [4,8,10,11]. We assessed the ability of chilled acinar cells to express fluorescent reporters under the control of the Amylase promoter in adenoviral vectors. For this purpose we used the Ad-Pamylase-nlsCre (Ad-pAmy-CRE) vector, which allows for expression of the CRE recombinase in cells with an active Amylase promoter, and the Ad-CMV-Lox-Stop-Lox-GFP, which allows for expression of the GFP reporter in cells that have previously expressed the CRE recombinase and excised the Lox-Stop-Lox sequence. Control or chilled exocrine cells cultured in suspension for 7 days were transduced at 37°C, and further cultured as adherent monolayers at 37°C for 5 days. The quantification of GFP expression was performed on day 12. We found that very few cells from control cultures were positive for the GFP reporter in these experiments (Figure 5A). On the contrary, chilled exocrine cell cultures showed many GFP-expressing cells after transduction (Figure 5B). Quantification of the mean amount of GFP+ cells per 4 cm^2^ of each condition showed a 4.75-fold increase in labelled cells counted in chilled (437 GFP+) compared with control (92 GFP+) cultures, and the average number of GFP+ cells per field was increased likewise (Figure 5C). This corresponded to labelling indices of <5% and 20–25% in control and chilled cultures respectively. These data indicate that the Amylase promoter remains in an active state in many chilled acinar cells for periods as long as 7 days.

### *De novo* protein synthesis is restricted in chilled acinar cells

**Figure 6:**
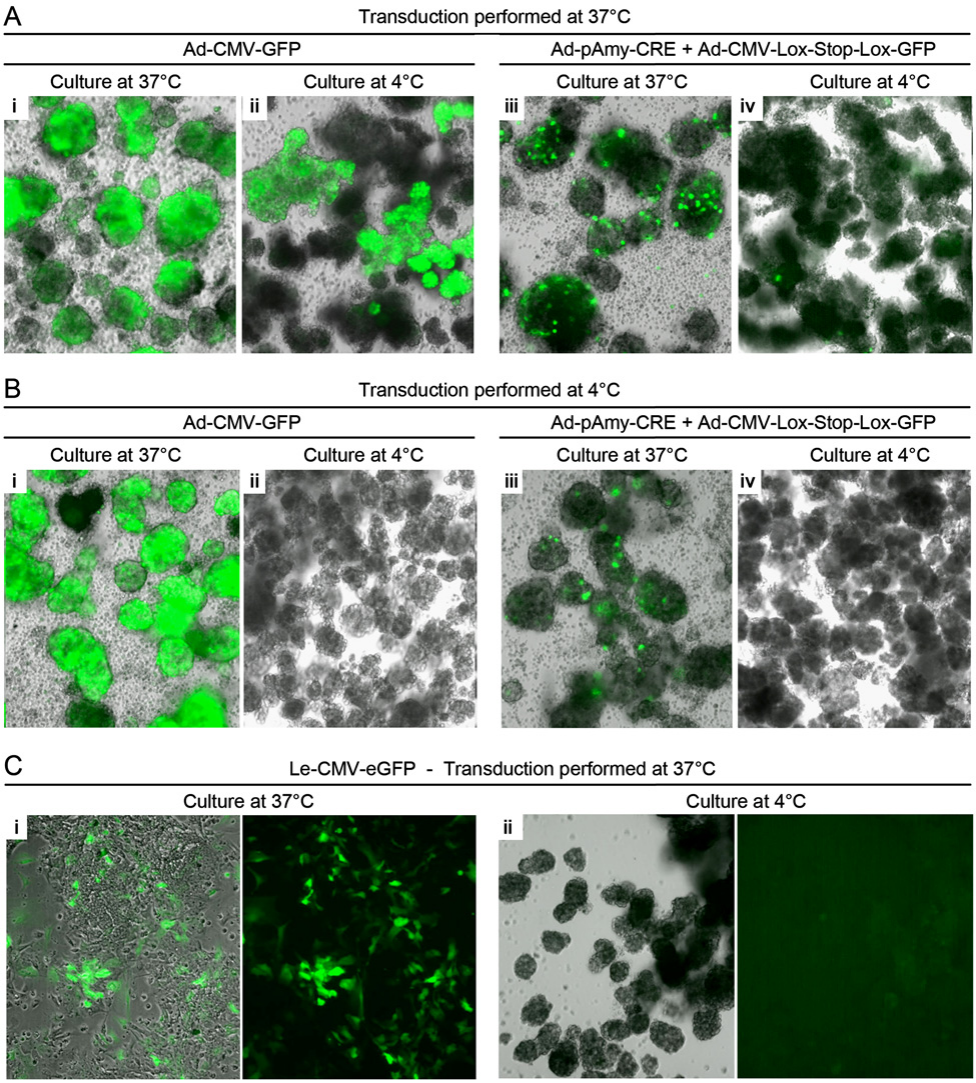
*De novo* protein synthesis is restricted in chilled cells (**A**) Representative images of exocrine cells transduced with the indicated vectors at 37°C on the day of isolation and cultured in suspension for 5 days at 37°C (**i** and **iii**) or at 4°C (**ii** and **iv**). (**B**) Representative images of exocrine cells transduced with the indicated vectors at 4°C on the day of isolation and cultured in suspension for 5 days at 37°C (**i** and **iii**) or at 4°C (**ii** and **iv**). Note the limited expression of GFP in cells cultured at 4°C compared with the ones at 37°C in both (A) and (B). (**C**) GFP signals in control (**i**) and chilled (**ii**) acinar cells transduced on day 0 at 37°C with Le-CMV-eGFP vectors and cultured for 5 days. Chilled culture is also associated with no GFP expression.

**Figure 7:**
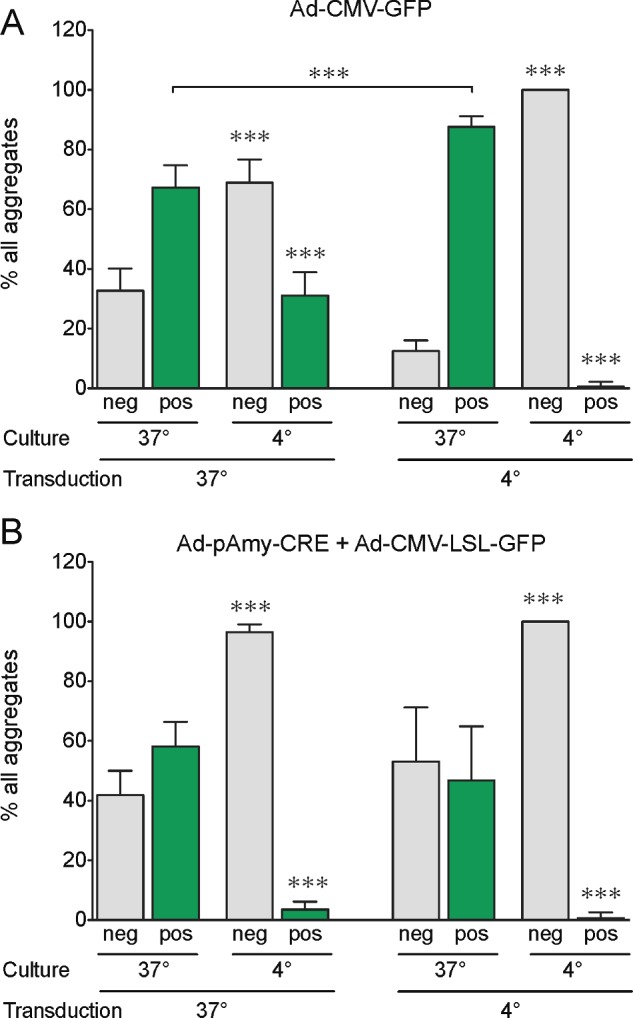
*De novo* protein synthesis is restricted in chilled cells (**A**) Estimation of GFP transgene expression in aggregates transduced at 37°C or at 4°C with Ad-CMV-GFP and cultured at 37°C or at 4°C for 5 days. (**B**) Estimation of GFP transgene expression in aggregates transduced at 37°C or at 4°C with a combination of Ad-pAmy-CRE and Ad-CMV-Lox-Stop-Lox-GFP and cultured at 37°C or at 4°C for 5 days. Note that there is practically no GFP+ aggregates when transduction and culture are performed at 4°C. The percentages of aggregates with GFP+ cells (pos) and those with GFP-negative cells (neg) were determined from fluorescent micrographs as the ones shown in Figures 6(A) and 6(B).

Considering the high levels of acinar transcripts and the demonstration of active Amylase promotor in 7-day chilled acinar cells, we further questioned whether chilling transduced acinar cells would increase their labelling efficiency. For this purpose, freshly isolated exocrine cells were transduced (Ad-CMV-GFP or Ad-pAmy-CRE with Ad-CMV-Lox-Stop-Lox-GFP) at 37°C or at 4°C on the day of isolation and further maintained in suspension at either of these temperatures for 5 days. We detected a much lower proportion of GFP+ cells in chilled cultures when compared with parallel 37°C cultures (Figures 6A and 6B; ii compared with i and iv compared with iii; Figures 7A and 7B). More specifically, there was practically no GFP+ cells detected when both transduction and culture were performed at 4°C. However, chilled transduction was efficient since nearly all clusters exposed to Ad-CMV-GFP at 4°C and further cultured at 37°C were positive for GFP to levels similar to the ones transduced at 37°C (Figures 6Ai, 6Bi and 7A). These findings therefore suggested that *de novo* protein synthesis did not occur in chilled exocrine cells. The limited protein synthesis in chilled cells could also be inferred by the fact that the combination of Ad-pAmy-CRE and Ad-CMV-Lox-Stop-Lox-GFP, which requires CRE protein expression and activity in excising the Lox-Stop-Lox site, resulted in lower level of GFP upon chilled transduction followed by culture at 37°C (Figures 6Aiii, 6Biii, 7A and 7B). Furthermore, lentiviral vector-based reporters (Le-CMV-eGFP) were transduced to isolated exocrine cells at 37°C and these were seeded in tissue culture plate for 5 days at 37°C or 4°C. As reported above, no GFP+ cells could be detected upon chilled culture whereas they were present in control cultures (Figure 6C). A similar transduction performed at 4°C resulted in no GFP expression in both 37°C and 4°C cultures (results not shown). Taken together, these findings suggest that there is no *de novo* protein synthesis in chilled acinar cells, and that acinar cell-specific proteins and transcripts detected in chilled cells (Figures 2 and 3) were not newly synthesized, but were the ones originally present on the day of isolation.

### Longer and chilled transduction improves the labelling index of exocrine cells

**Figure 8:**
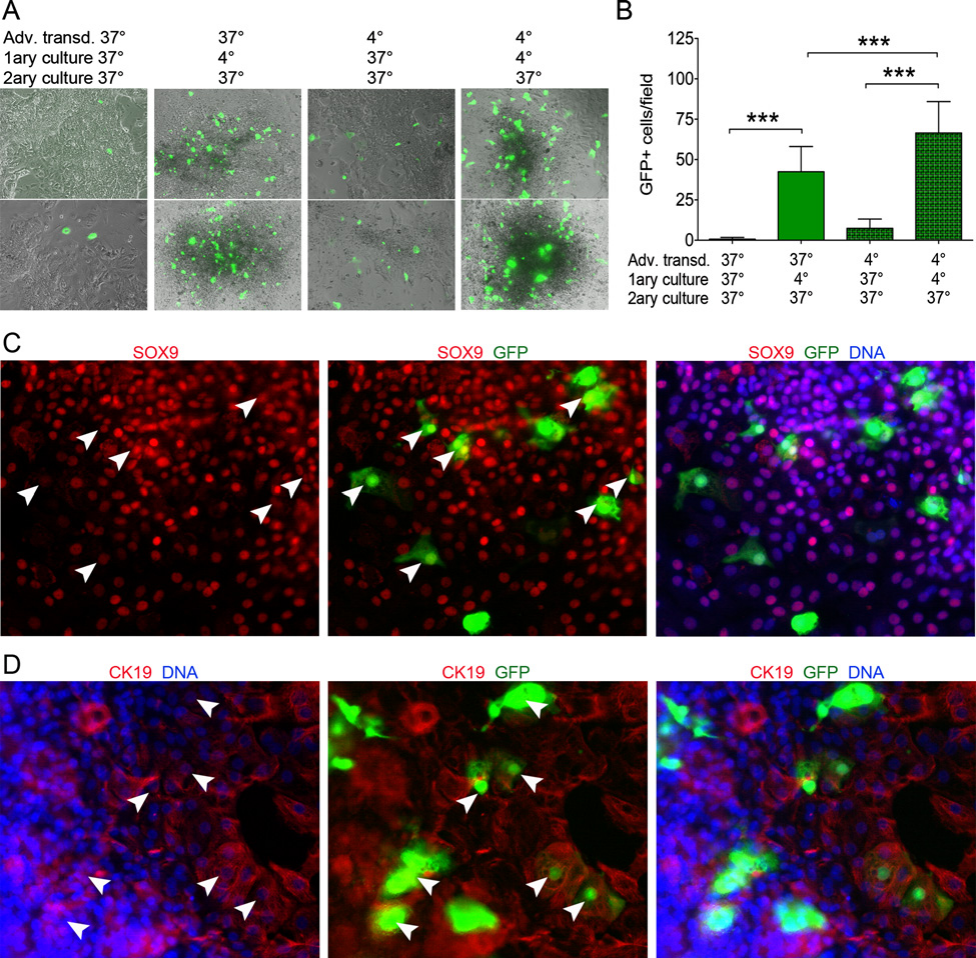
*De novo* protein synthesis is restricted in chilled cells (**A**) Representative images of exocrine cells transduced with Ad-pAmy-CRE and Ad-CMV-Lox-Stop-Lox-GFP vectors on the day of isolation, and cultured in suspension (primary culture, 5 days) then in tissue culture plates (secondary culture, 5 days) at the indicated temperatures. (**B**) Quantification of GFP expression at the end of secondary cultures for the four mentioned conditions. Note that chilled transduction and chilled primary culture are major determinants of higher GFP expression. (**C** and **D**) Immunofluorescence detection of the ductal markers SOX9 (C) and CK19 (D) in a subset of GFP+ cells at the end of secondary culture of cells transduced with Ad-pAmy-CRE and Ad-CMV-Lox-Stop-Lox-GFP on day 0. This confirms the acinar cell origin of these duct cells.

We finally considered the ability of chilled acinar cells to take up the transgenes and efficiently process them upon further culture at 37°C. Freshly isolated exocrine cells were transduced with Ad-pAmy-CRE and Ad-CMV-Lox-Stop-Lox-GFP at 37°C or 4°C, then cultured in suspension at 37°C or 4°C for 5 days, with medium refreshed every 2 days, therefore eliminating unbound viral particles. These 5-day cultured cells were then seeded in secondary cultures at 37°C for 5 additional days and analysed microscopically. In contrast with the control cultures transduced and cultured (primary and secondary) at 37°C, chilled transduction and/or chilled primary culture resulted in higher levels of GFP expression during secondary culture (Figures 8A and 8B). Interestingly, these initially transduced and chilled cells underwent the acino-ductal transdifferentiation mentioned above for secondary cultures, and we could trace the acinar origin of the transdifferentiated cells via the detection of the ductal markers SOX9 or CK19 in GFP+ cells (Figures 8C and 8D, Supplementary Figure S3).

In another set of experiments, exocrine cells were transduced overnight with Ad-pAmy-CRE and Ad-CMV-Lox-Stop-Lox-GFP and directly seeded in tissue culture plates at 37°C or at 4°C for 5 days. The longer exposure to viral vectors resulted in the detection of GFP expression in control cultures as well, and this was significantly improved by chilled transduction (overnight at 4°C). On the contrary, significantly few GFP+ cells were detectable in chilled cultures irrespective of the transduction conditions (Supplementary Figure S4), once again indicating limited protein synthesis at 4°C.

Taken together, our data indicate that adenoviral particles could be taken up by chilled cells but not processed due to the reduced metabolic activity, and that the persistently active Amylase promotor in chilled cells ensures an immediate and efficient CRE expression once the normal metabolic state is reconstituted by switching to 37°C.

## DISCUSSION

Islets of Langerhans isolated from deceased donor pancreata are currently transplanted in Type 1 diabetes patients who can then optimally achieve insulin-independence for long periods. Because the resulting normoglycaemia has been associated with delayed development or progression of diabetes complications, islet transplantation is considered a valuable therapy for Type 1 and advanced Type 2 patients. Unfortunately, the severe donor shortage limits its implementation to only a small fraction of selected patients. To circumvent this issue, alternative sources of insulin-producing β-cells must be sought. These might include pluripotent and multipotent stem cells, as well as non-endocrine cells from the pancreas with transdifferentiation potential such as the acinar and duct cells [1–3].

Acinar cells represent a good candidate because of their abundance in the pancreatic tissue, and because of their plasticity and their potential conversion to functional β-cells in *in vitro* and *in vivo* rodent models [9–16]. It is thus assumed that human acinar cells might become the cornerstone of regenerative medicine for diabetes in the future. In order to reach this goal, the transdifferentiation of human acinar cells into β-cells should be unambiguously demonstrated, preferentially using genetic lineage tracing techniques [25] in combination with endocrine phenotype induction by extracellular factors. To our knowledge, there is no study with such data available till date. To contribute to this caveat, we aimed at first evaluating the conditions that would improve the genetic labelling of human acinar cells, with a major focus on the *in vitro* maintenance of the acinar phenotype in primary cultures. These conditions would be implemented in future experiments to demonstrate the acinar to β-cell transdifferentiation, either by means of xenograft models or of *in vitro* differentiation approaches involving sequential and combined supplementation of small molecules and growth factors.

Currently, a major limitation to the demonstration of this conversion is the difficulty to use long-term viral vector-based labelling methods on acinar cells, due to their phenotypic instability in culture and rapid loss of expression of marker genes [4,7,26]. Cre/Lox technology under the control of an acinar-specific promoter therefore does not offer much improvement of the labelling efficiency since the acinar-specific promoter activity (driving CRE) is quickly and drastically down-regulated as soon as the acinar cells are put in culture. Whether acinar cell phenotype could be maintained *in vitro* has already been a matter of laboratory investigation. Nicotinamide or sodium butyrate was shown to maintain specific protein expression in rodent acinar cells [7], but unfortunately these treatments inhibit viral infection (unpublished observations). Phenotype preservation was shown in fragments or Matrigel-embedded acini [18,20], but an efficient labelling is not possible in these thick specimen. In the previous study, Coutts et al. [21] showed that hypothermic storage could preserve the human acinar tissue morphology and secretory capacity. We reproduced the findings of Coutts et al., and further showed that acinar cell transcripts and proteins remain detectable at high levels up to day 10 whereas they are practically cleared off in control cultures. That expression of the ductal marker *CK19* remained similar to basal levels during the 5 days of chilled culture is an indication of a delayed or restrained acino-ductal transdifferentiation in this condition. Coupled with the persistently active promotor of acinar cell-specific markers such as Amylase, these findings provide strong evidence that the chilled condition could be exploited to delay acinar cell phenotype loss *in vitro* and therefore to use genetic lineage tracing methods that are known to be inefficient for acinar cells in cultures performed at 37°C. We demonstrated this by the use of a combination of Ad-pAmy-CRE and Ad-CMV-Lox-Stop-Lox-GFP, which clearly revealed major Amylase promotor-driven recombination processes occurring upon chilled transduction and/or chilled culture. Our study identifies the overnight transduction at 4°C followed by seeding at 37°C as the best condition that results in a labelling efficiency of nearly 30%. We are confident that the acinar cell-specific labelling achieved with our methods will be valuable in future experiments aiming at lineage-tracing β-cell neogenesis in human exocrine cell cultures or in human acinar cell xenograft models. For instance, these methods could be combined with the protocol developed by Lima et al. for the differentiation of human exocrine pancreatic tissue toward β-like cells, or with those recently developed for the differentiation of human pluripotent stem cells into functional insulin-producing cells [26–28]. We already provided in the present study a proof of concept regarding this issue: the acinar origin of transdifferentiated cells in secondary cultures was evidenced via the detection of the ductal markers CK19 or SOX9 in GFP+ cells. Therefore, the same may ensure tracing of human β-cell neogenesis in the future.

Besides providing a method for efficient acinar cell labelling, our study also suggested restriction of *de novo* protein synthesis in chilled culture, as assessed by the expression of GFP reporter either in constitutive (Ad-CMV-GFP, Le-CMV-GFP) or conditional (Ad-pAmy-CRE + Ad-CMV-Lox-Stop-Lox-GFP) vectors. It was actually this observation that prompted us to perform a longer transduction in chilled condition, which fortunately improved acinar cell labelling. In addition, the absence of *de novo* protein synthesis indicates that chilled acinar cells enter in a resting metabolic state and that transcripts and proteins present in isolated exocrine cells can be very well preserved by cold storage for few days. This preservation can be seen as a continuation of the cold preservation that is applied to human pancreas before islets isolation [29,30]. Interestingly, we showed that the Amylase promotor, which is very rapidly silenced during acinar cell culture at 37°C, was maintained active in chilled cultures and was responsible for abundant acinar cell labelling upon transduction with Ad-pAmy-CRE and Ad-CMV-Lox-Stop-Lox-GFP vectors. Such an improvement of labelling by longer and chilled transduction suggests the synergy between two phenomena: first, the increased viral particle uptake by many more cells than during the 3 h transduction; and second, the meanwhile maintenance of active Amylase promoter to ensure efficient expression of CRE recombinase upon culture at 37°C. It remains unclear whether the active Amylase promotor contributes anyhow to the abundant transcripts of this gene detected during chilled culture. Similarly, the RT-PCR assays that we performed cannot inform on whether active transcription of *CK19* and *NGN3* occurred in chilled cultures or whether the increased levels were associated with relative enrichment in particular cell populations.

Our data show a clear discrepancy between the outcomes of exocrine cell transduction and culture at different temperatures. Although we assume this observation to be largely affected by the difference in temperature between both settings, we cannot exclude the contribution of other non-controlled parameters such as the levels of O_2_ and CO_2_ that are very likely to be different too. It would be interesting in the future to control these parameters and evaluate the effect of culturing at intermediate temperatures (15–20°C) where we expect the metabolism to be just moderately limited. In this case, particular attention would be paid to the transcription of the pro-endocrine gene *NGN3*.

## Abbreviations

Ad: adenovirus
CK19: cytokeratin 19
CNTF: ciliary neurotrophic factor
EGF: epidermal growth factor
(e)GFP: (enhanced) green fluorescent protein
Le: lentivirus
MOI: multiplicity of infection
Ngn3: neurogenin 3
PDX1: pancreatic and duodenal homeobox 1
PTF1a: pancreas transcription factor 1 subunit alpha
qPCR: quantitative polymerase chain reaction
RT-PCR: reverse transcription polymerase chain reaction
